# Evaluation of Clinical Variables Affecting Myocardial Glucose Uptake in Cardiac FDG PET

**DOI:** 10.3390/diagnostics14161705

**Published:** 2024-08-06

**Authors:** Yeongjoo Lee, Jaehyuk Jang, Sungmin Lim, Sae Jung Na

**Affiliations:** 1 Division of Nuclear, Medicine Department of Radiology, Uijeongbu St. Mary’s Hospital, College of Medicine, The Catholic University of Korea, Seoul 11765, Republic of Korea; soap2222@msn.com; 2 Division of Cardiology, Department of Internal Medicine, Uijeongbu St. Mary’s Hospital, College of Medicine, The Catholic University of Korea, Seoul 11765, Republic of Korea; let87@naver.com (J.J.); mdsungminlim@catholic.ac.kr (S.L.)

**Keywords:** cardiac PET, FDG, BMI, obesity, myocardial glucose uptake

## Abstract

Purpose: Cardiac 2-deoxy-2-[F-18]fluoro-D-glucose positron emission tomography (FDG PET) is widely used to assess myocardial viability in patients with ischemic heart disease. While sufficient glucose uptake is a prerequisite for accurate interpretation of cardiac viability, there are a lack of data on which clinical variables have the most significant impact on myocardial glucose metabolism. Therefore, this study was designed to evaluate several clinical variables that could affect myocardial glucose metabolism. Methods: A total of 214 consecutive cases were retrospectively enrolled in this study. All subjects received 250 mg of acipimox and underwent glucose loading as preparation for cardiac FDG PET/CT. Three-dimensional regions of interest (ROIs) were drawn on PET/CT fusion images. Myocardial glucose uptake ratio (MGUR = SUVmax of LV myocardium/SUVmean of liver) was then calculated. Multiple clinical variables including body mass index (BMI), blood glucose levels at different times, administered insulin dosage, lipid profiles, and ejection fraction were measured and analyzed for correlation with myocardial glucose uptake. After dichotomizing the subjects based on a BMI of 25, each group’s MGUR was compared. Results: Myocardial uptake showed significant correlations with BMI (r = −0.162, *p* = 0.018), HbA1c (r = −0.150, *p* = 0.030), and triglyceride levels (r = −0.137, *p* = 0.046). No other clinical variables showed a significant correlation with myocardial glucose uptake. After multiple linear regression analysis, BMI (*p* = 0.032) and HbA1c (*p* = 0.050) showed a correlation with MGUR. In group analysis, after dividing patients based on BMI, the obese group showed significantly lower myocardial uptake than the non-obese group (3.8 ± 1.9 vs. 4.4 ± 2.1, *p* = 0.031). Conclusions: Among several clinical variables, BMI and HbA1c levels were related to myocardial glucose uptake. A prospective study would be needed to examine whether a protocol that additionally considers BMI and HbA1c levels is necessary for the current cardiac FDG PET protocol.

## 1. Introduction

The prevalence of ischemic heart disease is rising worldwide, driven primarily by an aging population [1]. A recent report has indicated that ischemic heart disease affects approximately 126 million individuals worldwide, accounting for 1.72% of global population [2]. For cardiologists facing an increasing number of patients with ischemic heart disease, accurately evaluating the extent and severity of viable myocardial tissue is of utmost importance. Identifying viable myocardial tissue enables clinicians to anticipate functional recovery after revascularization. Numerous previous reports have indicated that identifying myocardial viability can benefit patients with ischemic heart disease undergoing revascularization [3,4,5]. Conversely, if the tissue is non-viable, it can reduce unnecessary invasive procedures, benefiting patients both medically and economically. The assessment of myocardial viability has been a challenging issue for clinicians, as variable degrees of ischemia can occur at different time points, resulting in the coexistence of different phases of acute, subacute, and chronic conditions.

The Food and Drug Administration (FDA) has approved cardiac 2-deoxy-2-[F-18]fluoro-D-glucose positron emission tomography (FDG PET) imaging as the imaging technique for assessing myocardial viability [6]. Cardiac FDG PET has been widely used for viability assessment and proven to be beneficial for patients’ recovery in previous studies [7,8]. A notable example was the large randomized study called ‘Positron emission tomography and recovery following revascularization (PARR-2)’, which demonstrated a significant advantage in terms of cardiac death, myocardial infarction, and cardiac hospitalization when cardiac FDG PET was performed compared to cases where it was not conducted [9].

The current imaging guidelines from the American Society of Nuclear Cardiology (ASNC) and the procedure standards from the Society of Nuclear Medicine and Molecular Imaging (SNMMI) in 2016 focus on the metabolism of glucose over fatty acids [10]. Patient preparation is determined based solely on blood glucose level. For optimal myocardial glucose uptake, the current guideline recommends maintaining a basal glucose level of approximately 100–140 mg/dL (5.55–7.77 mmol/L) at the time of FDG injection and adjusting insulin dosage based on blood glucose level after glucose loading. However, some patients achieve acceptable imaging quality with the same amount of insulin administration, while others fail to achieve sufficient myocardial glucose uptake despite receiving an adequate amount of insulin. An overestimated insulin dose may cause unexpected hypoglycemia that requires immediate glucose replacement followed by careful monitoring. This situation may require additional delayed images or even necessitate re-examination.

We assume that various clinical factors including baseline blood glucose levels might influence myocardial glucose metabolism. A previous report has suggested that both peripheral insulin resistance and increasing age may independently affect FDG uptake in the myocardium [11], although other clinical features related to glucose metabolism need to be verified. Therefore, this study aimed to investigate correlations of various clinical factors including baseline blood glucose levels with myocardial glucose uptake in patients with ischemic heart disease undergoing cardiac FDG PET.

## 2. Materials and Methods

### 2.1. Subjects

Medical records of patients with ischemic cardiopathy who underwent cardiac FDG PET/CT at Uijeongbu St. Mary’s Hospital between May 2018 and November 2022 were retrospectively reviewed. All patients had previous records of coronary angiography of chronic total occlusion in at least one of the cardiac coronary vessels. They were referred to our department for cardiac FDG PET/CT to assess myocardial viability. Patients with inflammatory conditions such as infective endocarditis and pericarditis were excluded. A total of 214 consecutive cases of cardiac FDG PET/CT were enrolled in this cohort.

Clinical parameters such as patient’s age, sex, height, weight, fasting blood sugar test (BST), BST after glucose loading, and BST at FDG injection were recorded. Patients who take diabetic medication were recorded. If necessary, regular insulin was administered based on blood sugar level after oral glucose loading and its dosage was recorded. Laboratory data including hemoglobin A1c, total cholesterol, low-density lipoprotein (LDL), high-density lipoprotein (HDL), triglyceride, troponin T, creatinine (Cr), and creatine kinase-myoglobin binding (CK-MB) were also recorded. All lab data were acquired within a month prior to cardiac FDG PET, with an average of 2.8 ± 13.6 days from PET. Ejection fraction was measured and recorded based on the echocardiogram taken within a month of the cardiac FDG PET. Results from the coronary angiography conducted prior to cardiac FDG PET were also recorded.

### 2.2. Image Acquisition and Measurement

Cardiac FDG PET/CT images were acquired using a single PET/CT scanner (40 TruePoint with True V, Siemens Medical Solutions, Knoxville, TN, USA) following administration of 370 MBq (10.4 ± 0.6 mCi) of FDG. All patients were administered 250 mg of acipimox, a nicotinic acid derivative known to reduce plasma free-fatty-acid levels and enhance myocardial glucose uptake. Regular insulin was administrated as needed, based on blood sugar level prior to exam, in accordance with the in-hospital protocol (Supplementary Table S1).

**Figure 1:**
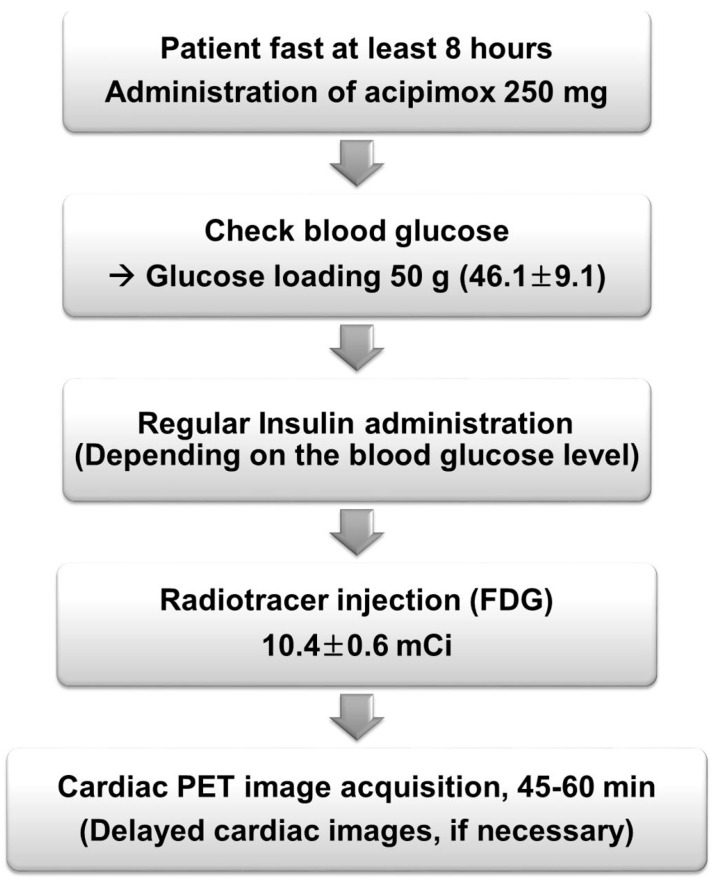
Flow chart of the patient preparation and cardiac FDG PET.

A CT scan was performed for attenuation correction before PET acquisition. Images of cardiac FDG PET were obtained in a single bed for fifteen minutes. After injection of radiotracer, images were taken between 45 and 60 min with patients in a supine position and their arms raised bilaterally. The time interval between radiotracer injection and scanning was recorded for analysis. Each cardiac PET image was reconstructed with a 256 × 256 matrix and a 3 mm Gaussian filter. Attenuation correction was performed immediately after CT transmission imaging. An iterative reconstruction algorithm of 3D ordered-subset expectation maximization (OSEM) was applied with 6 iterations and 16 subsets. A flowchart of patient preparation and the imaging process is summarized in Figure 1.

**Figure 2:**
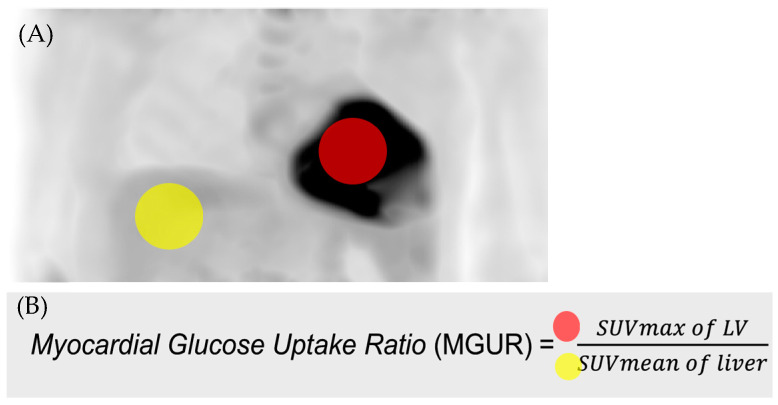
(**A**) 3 cm-sized spheres of Region of Interest (ROI) were drawn in the wall of the left ventricle (LV, red circle) and liver (yellow circle), respectively. (**B**) Myocardial glucose uptake ratio (MGUR) was calculated as SUVmax of LV divided by SUVmean of liver.

For analysis of myocardial uptake, 3 cm-sized spheres of the Region of Interest (ROI) were drawn in the wall of the left ventricle (LV) and liver, respectively. The maximum standardized uptake value (SUVmax) of LV and the mean standardized uptake value (SUVmean) of liver were measured and used to calculate myocardial glucose uptake ratio (MGUR). Siemens-TrueD 2009B(VC60) software (Siemens Medical Solutions, Knoxville, TN, USA) was used for analysis. An example of drawing the ROI is presented in Figure 2.

### 2.3. Data Analysis

The Pearson correlation test and linear regression analysis were applied to examine linear associations of clinical factors with MGUR. Fitting results of linear regression were compared, to test whether slopes and intercepts differed significantly. For factors showing significance with a *p*-value of up to 0.2 in the univariable linear regression analysis, additional multivariable analysis was conducted using the stepwise method. According to the Asia–Pacific criteria of the World Health Organization guidelines, obesity in East Asian adults is defined as a Body Mass Index (BMI) of 25 kg/m^2^ or greater. For group analysis, subjects were dichotomized into an obese group (BMI ≥ 25) and a non-obese group (BMI < 25) based on their BMI. Binary groups dichotomized by BMI were then compared in terms of their clinical factors including age, gender, height, weight, BMI, ejection fraction, fasting BST, loading BST, injection BST, total cholesterol, triglyceride, HDL, LDL, troponin T, Cr, CK-MB, and HbA1c. Group comparisons were made after trichotomizing the patients with cutoff value of BMI of 25 and 30, using one-way analysis of variance (ANOVA). Additionally, a comparison of MGUR between diabetic and non-diabetic patients was performed. Student’s *t*-test was employed to compare clinical factors between binary groups. Statistical analyses were performed using the Statistical Package for the Social Sciences (SPSS) version 24.0 (IBM Corporation, Armonk, NY, USA). A *p*-value of less than 0.05 was considered statistically significant.

## 3. Results

### 3.1. Patient Characteristics

A total of 214 patients were included in this study for analysis. The average age of all included patients was 64.8 (±11.4) years, ranging from 39 to 87 years. Of these, 101 patients were previously diagnosed with diabetes, while 113 were not diagnosed as diabetic at the time of examination. The mean (±SD) BMI was 24.8 ± 3.5. One hundred and eighty-nine (88.9%) patients had patent left main (LM) coronary artery, while twenty-five (11.7%) patients had LM stenosis (51.0% ± 27.3). Forty-two (19.6%) patients had a one-vessel disease (VD), 87 (40.7%) patients had a two-vessel disease, and 84 (39.9%) patients had a three-vessel disease. One patient had a prior history of Coronary Artery Bypass Grafting (CABG). All subjects underwent an echocardiogram prior to cardiac PET, revealing an average ejection fraction of 47.3% (±12.7). Sixty patients out of 214 (60/214, 28.0%) had EF less than 40%. The mean fasting blood sugar test (BST) after at least 8 h of fasting (nil per os, NPO) was 121.7 (±32.9) mg/dL, while the BST after glucose loading was 200.2 (±54.2) mg/dL. Of all patients, 201 underwent an oral glucose loading of 50 g and 15 had a loading of 25 g. BST at the time of FDG injection was 194.5 ± 49.0 mg/dL. On average, 2.9 (±2.1) IU of insulin was administered to 103 patients before cardiac PET, based on their basal glucose levels. The average HbA1c level was 6.7 ± 1.5% and the time between the cardiac PET and laboratory data was 2.8 ± 13.6 days. The average injection-to-image time was 52.8 ± 8.0 min. The SUVmax of the left ventricle was 9.2 (±3.6). The SUVmean of the liver was 2.4 (±1.2) and the average myocardial glucose uptake ratio (MGUR) calculated was 4.2 (±2.0). Other detailed patient characteristics are summarized in Table 1. Non-diabetic patients (*n* = 113) showed slightly higher MGUR than diabetic patients (*n* = 101) (4.4 ± 1.8 vs. 3.8 ± 2.1, *p* = 0.034).

### 3.2. Correlation Analysis with MGUR

**Figure 3:**
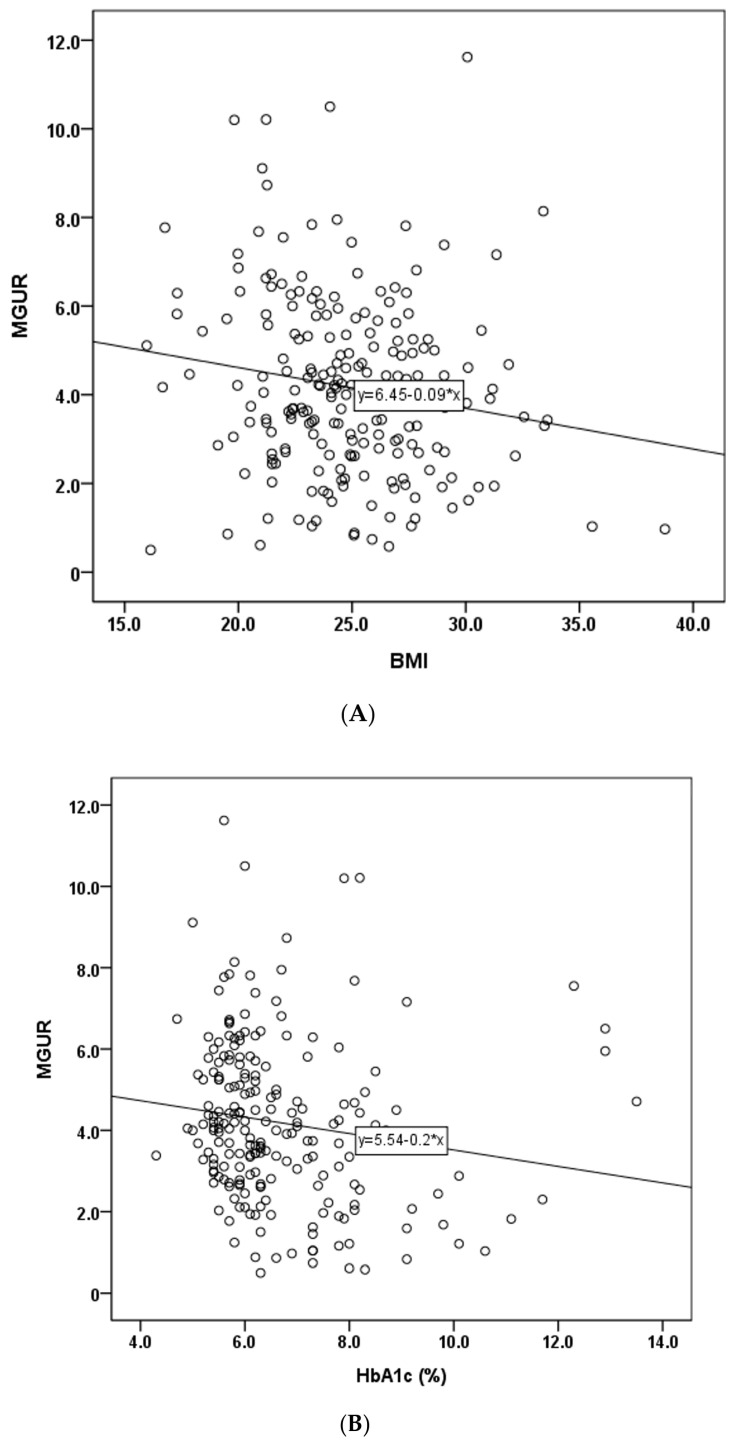

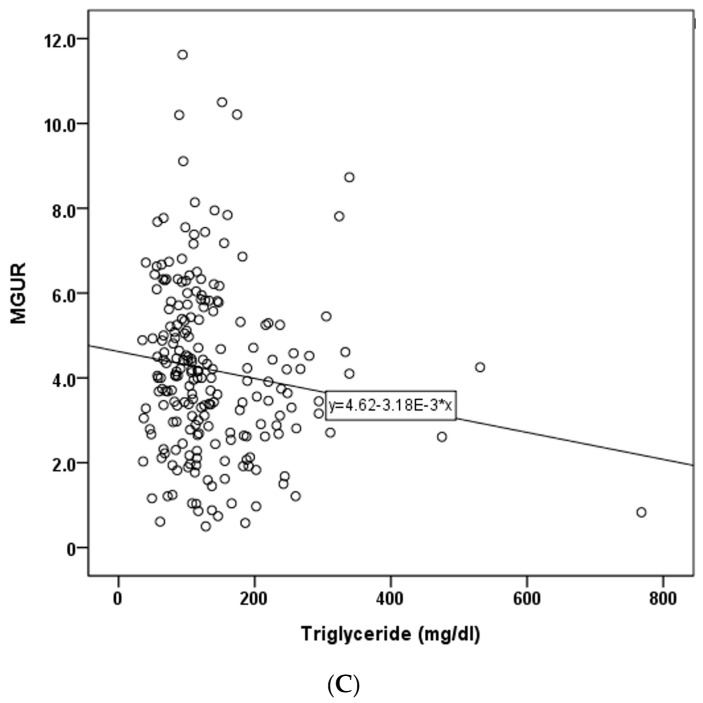
Scatter plots that showed correlation with MGUR (myocardial glucose uptake ratio). (**A**) BMI (Body Mass Index), (**B**) HbA1c (%), (**C**) triglyceride (mg/dL).

Correlation analysis of clinical factors and MGUR revealed significant negative correlations of MGUR with BMI (r = −0.162, *p* = 0.018), HbA1c (r = −0.150, *p* = 0.030), and triglycerides (r = −0.137, *p* = 0.046). Scatter plots of these significant factors are depicted in Figure 3 (panels A, B, and C). No significant correlations were observed between MGUR and other clinical factors such as age, height, weight, ejection fraction (EF), troponin T, CK-MB, fasting BST, loading BST, injection BST, insulin, creatinine, total cholesterol, HDL, or LDL. Results of univariable and multivariable linear regression analysis of clinical factors with MGUR are presented in Table 2. BMI (*p* = 0.032) and HbA1c (*p* = 0.050) were found to be independently correlated with MGUR. The regression model accounted for 21.0% of the variance in MGUR, as indicated by an R-squared value of 0.210. The adjusted R-squared value of the model was 0.035. The F-statistic for the model was 4.775, with a corresponding *p*-value of 0.009, indicating an overall statistical significance of the regression model.

### 3.3. Group Analysis According to BMI

After dichotomizing subjects based on their BMI, the non-obese group (BMI < 25) consisted of 119 subjects (male: female = 99:20), while the obese group (BMI ≥ 25) included 95 subjects (male:female = 79:16). The non-obese group had a significantly higher MGUR than the obese group (4.4 ± 2.1 vs. 3.8 ± 1.9, *p* = 0.036). Regarding other clinical data, the non-obese group exhibited a significantly higher HDL level than the obese group (43.1 ± 12.7 vs. 38.9 ± 8.4, *p* = 0.006). The two groups showed no significant differences in age, height, ejection fraction (EF), troponin T, CK-MB, fasting BST, loading BST, injection BST, insulin, HbA1c, creatinine, total cholesterol, triglycerides, or LDL. Detailed comparisons of clinical factors between the two groups are summarized in Table 3. No significant MGUR difference was observed after trichotomizing patients according to BMI of 25 and 30 (BMI < 25 vs. 25 ≤ BMI < 30, *p* = 0.77; 25 ≤ BMI < 30 vs. 30 ≤ BMI, *p* = 0.82; BMI < 25 vs. 30 ≤ BMI, *p* = 0.79) (Supplementary Table S2 and Figure S1).

## 4. Discussion

As the prevalence of ischemic heart disease increases, the assessment of myocardial viability is increasingly important. Given the challenge of confirming myocardial viability through coronary angiography in chronic total occlusion (CTO), imaging modalities such as cardiac magnetic resonance (CMR) and cardiac FDG PET are considered as preferred methods for viability assessment. CMR offers high spatial resolution, enabling detection of size and assessment of the transmural extent of myocardial scar. It also provides structural information without the risk of radiation exposure. Late gadolinium enhancement of less than 50% of wall thickness is regarded as a viable tissue. CMR shows comparatively high sensitivities and specificities, and substantial evidence base [12,13]. However, CMR is limited for patients with pacemakers or internal cardiac defibrillators, and gadolinium-based contrast is contraindicated for those with reduced renal function (eGFR < 30 mL/min/1.73 m^2^) or severe claustrophobia.

In comparison, cardiac FDG PET has emerged as a significant functional imaging tool for predicting left ventricular (LV) functional recovery [14]. Regions in which cardiac FDG uptake is increased relative to a perfusion defect are called ‘perfusion–metabolism mismatch’. They represent viable hibernating myocardium. With accumulated clinical data, the role of cardiac FDG PET has been considered as the reference standard for myocardial viability. Schinkel et al. have reported that weighted mean sensitivity and specificity of cardiac FDG PET are 92% and 63%, respectively [3]. Evaluating the hibernating myocardium has significance in identifying patients for whom revascularization enhances prognosis. Kandolin et al. have suggested that viability assessment should be performed for high-risk patients with comorbidity before revascularization [15]. Multiple other studies have shown a connection between viability identified through cardiac FDG PET and different clinical outcomes [16,17,18,19].

While viability assessment using cardiac FDG PET is known to be beneficial, achieving proper patient preparation is not always readily achievable, due to the ‘dual metabolism’ of myocardial tissue involving both fatty acids and glucose. The myocardium exhibits ‘metabolic plasticity’, allowing it to utilize various energy sources even under different metabolic conditions, including ischemia. Typically, it prefers fatty acid metabolism due to its higher carbon content per molecule, resulting in more ATP production. For instance, palmitate and oleate as fatty acid metabolites contain 16 and 18 carbons, respectively, while glucose contains only 6 carbons. However, in cells that are viable but at risk, there is an increase in FDG uptake due to a transition towards anaerobic metabolism and a preference for glucose metabolism over fatty acid metabolism [11]. The duration of fasting and the level of endogenous insulin at the time of radiotracer injection can significantly influence myocardial glucose uptake. Thus, accumulated clinical experience is needed to optimize image quality.

This study aimed to evaluate several clinical variables that might influence myocardial glucose metabolism in patients with ischemic heart disease. Each clinical factor, including BMI, HbA1c, and triglyceride, demonstrated a negative correlation with myocardial glucose uptake. In group comparison, the obese group demonstrated significantly lower myocardial uptake than the non-obese group, suggesting altered myocardial metabolism in obesity. This aligns with previous reports indicating an association between obesity and increased myocardial fatty acid metabolism at the expense of glucose metabolism [20]. The decrease in glucose uptake in obese patients can be partially explained by excessive uptake and utilization of fatty acids. This process can inhibit full glucose oxidation more than glycolysis and glucose uptake [21]. Previous reports have also demonstrated excessive delivery and utilization of fatty acids to the myocardium using C-11 palmitate PET [22,23]. Herrero et al. have reported that patients with type I diabetes exhibit higher myocardial fatty acid uptake, utilization, and oxidative stress, but lower glucose utilization than normal controls [24]. Previous studies have reported that subjects undergoing bariatric surgery show reduced fatty acid metabolism [25] but increased glucose metabolism [26]. These findings imply that the degree of obesity might have an impact on myocardial glucose uptake at an individual level.

A previous study using mouse models showed a significant increase in myocardial cannabinoid type 1 receptor (CB1-R) expression in advanced obesity compared to normal weight controls [27]. It appears that activation of the endocannabinoid system in obesity could stimulate the expression and upregulation of myocardial CB1-R, which could reflect altered metabolism. Hence, we could assume that obesity somehow contributes to altered myocardial metabolism via excessive delivery of free fatty acids and triglycerides.

Even though HDL did not show statistically significant results from a relatively strict statistical perspective, it is noteworthy that there was a weak positive correlation between MGUR and HDL. Furthermore, in the dichotomized group comparison between the non-obese and obese groups, HDL, rather than LDL, showed differences, as well as MGUR. This suggests that metabolism transit in the myocardium may be more affected by HDL, whose main function is to efflux lipids from peripheral tissues and transport them to the liver. Future studies with varied dyslipidemia conditions are needed to verify the exact impact of lipoproteins on myocardial glucose metabolism.

Additionally, our results showed there is no significant correlation between ejection fraction and myocardial glucose metabolism. Some authors have previously reported contradictory results, suggesting that myocardial glucose metabolism is reduced in heart failure patients compared to normal healthy controls, due to increased insulin resistance in myocytes [28,29]. However, those studies were conducted with different patient preparation methods, which are quite different from our protocol. We aimed to maximize physiological glucose uptake in viable myocardium by administering lipid-lowering agents, along with deliberate glucose loading followed by sufficient fasting, and individualized insulin administration. We believe that this preconditioning resulted in sufficient glucose uptake in some of the viable myocardial tissue in heart failure patients, thus demonstrating increased SUVmax.

In diabetic patients, it is challenging to determine how diabetes-related clinical factors affect the myocardium when performing cardiac FDG PET. This difficulty arises from the variety of diabetes medications, as well as individual variations in disease duration, medical compliance, and inaccuracies in history taking. Although the pathophysiology of type I and type II diabetes may differ, it is anticipated that the overall mechanism of glucose metabolism in each type of diabetes could be similar. Our presented study considered HbA1c levels at the time of cardiac FDG PET as an alternative objective value, as many non-diabetic patients might have unknown insulin resistance, while diabetic patients on medication might have well-controlled blood glucose levels at the time of radiotracer injection. The significance of HbA1c in the diagnosis and prognosis of diabetic patients is well documented in a previous report [30].

This study has some limitations. Firstly, it was a single-center retrospective study. Secondly, not all patients had previous perfusion images to investigate perfusion–metabolism mismatch. Instead, all patients underwent coronary angiography and had a history of complete total occlusion in their coronary vessels. Thirdly, while SUVmax is a convenient value for tumor metabolism, its representation of myocardial metabolism is debatable. Although the SUVmax value does not fully represent the entire spectrum of cardiac metabolism, it can provide insights into glucose uptake and utilization. Fourthly, although there is a mild negative linear correlation between BMI and MGUR, we did not find a statistically significant decrease in myocardial glucose uptake between the extremely obese group (BMI ≥ 30) and moderately obese patients (25 ≤ BMI < 30). We assume the reason is that the number of patients in the extremely obese group is much smaller compared to the other groups, so a few outliers may have negatively affected the statistical results. A future multicenter prospective study with a large cohort is necessary to ascertain the relationship between glucose metabolism and clinical factors, to determine optimal conditions for maximizing myocardial uptake.

In conclusion, BMI and HbA1c levels were related with myocardial glucose uptake. A prospective study may be needed to examine whether a protocol that additionally considers BMI and HbA1c levels is necessary for the current cardiac FDG PET protocol.

## Figures and Tables

**Table 1:** Patient characteristics (*n* = 214).

Characteristics	Value
Man/Woman	178/36
Non-diabetic/Diabetic	113/101
Age (years)	64.8 ± 11.4
Height (cm)	164.8 ± 8.8
Weight (kg)	67.5 ± 12.2
BMI (kg/m^2^)	24.8 ± 3.5
Coronary (artery) disease	
Left main artery disease	
Patent	189 (88.3%)
Stenosis	25 (11.7%)
1VD ^1^	42 (19.6%)
2VD	87 (40.7%)
3VD	84 (39.3%)
Ejection Fraction (%)	47.3 ± 12.7
Cardiac markers	
Troponin (ng/mL)	0.88 ± 2.14
CK-MB ^2^ (ng/mL)	21.0 ± 55.5
HbA1c ^3^ (%)	6.7 ± 1.5
Creatinine (mg/dL)	1.4 ± 1.5
Lipid profile (mg/dL)	
Total cholesterol	154.2 ± 49.2
Triglyceride	136.9 ± 86.5
HDL ^4^	41.2 ± 11.2
LDL ^5^	88.6 ± 45.7
Blood Sugar Test (mg/dL)	
fasting BST ^6^	121.7 ± 32.9
BST after glucose loading	200.2 ± 54.2
BST at FDG injection	194.5 ± 49.0
Injected insulin (IU)	2.9 ± 2.1
Injection-to-image time (minutes)	52.8 ± 8.0
SUVmax of left ventricle	9.2 ± 3.6
SUVmean of liver	2.4 ± 1.2
Myocardial Glucose Uptake Ratio	4.2 ± 2.0

^1^ VD (vessel disease); ^2^ CK-MB (creatinine kinase-MB); ^3^ HbA1c (glycated hemoglobin); ^4^ HDL (high-density lipoprotein); ^5^ LDL (low-density lipoprotein); ^6^ BST (blood sugar test).

**Table 2:** Univariable and multivariable linear regression analyses of clinical factors with myocardial glucose uptake ratio (MGUR).

Variables	Univariable			Multivariable	
	R^2^	β (SE ^6^)	*p*-Value	β (SE)	*p*-Value
Age	0.11	−0.019 (0.012)	0.127	−0.126	0.068
Height	0.002	0.010 (0.016)	0.525		
Weight	0.012	−0.018 (0.011)	0.107	0.021	0.852
BMI	0.026	−0.092 (0.038)	0.018 *	−0.084 (0.039)	0.032 *
Ejection Fraction	0.007	−0.013 (0.011)	0.226		
Troponin T	0.000	−0.005 (0.065)	0.935		
CK-MB ^1^	0.000	0.000 (0.002)	0.961		
Fasting BST ^2^	0.000	−0.001 (0.004)	0.821		
BST after glucose loading	0.020	−0.001 (0.003)	0.561		
BST at FDG injection	0.008	−0.004 (0.003)	0.186	−0.046	0.546
Insulin	0.003	0.049 (0.067)	0.461		
HbA1c ^3^	0.022	−0.202 (0.092)	0.030 *	−0.182 (0.092)	0.050 *
Creatinine	0.000	0.017 (0.091)	0.850		
Total cholesterol	0.005	0.003 (0.003)	0.319		
Triglyceride	0.019	−0.003 (0.002)	0.046 *	−0.111	0.108
HDL ^4^	0.018	0.024 (0.012)	0.052	0.075	0.298
LDL ^5^	0.008	0.004 (0.003)	0.185	0.069	0.314

* Statistically significant. ^1^ CK-MB (creatinine kinase-MB); ^2^ BST (blood sugar test); ^3^ HbA1c (glycated hemoglobin); ^4^ HDL (high-density lipoprotein); ^5^ LDL (low-density lipoprotein); ^6^ SE (standard error).

**Table 3:** Comparison of variables between the non-obese and obese groups (*n* = 214).

Variables	Non-Obese Group (BMI ^1^ < 25, *n* = 119)	Obese Group (BMI ≥ 25, *n* = 95)	*p*-Value
Weight	61.3 ± 9.2	75.3 ± 11.0	<0.001 *
MGUR ^2^	4.4 ± 2.1	3.8 ± 1.9	0.036 *
Age (year)	65.1 ± 10.9	64.3 ± 12.0	0.598
Height	165.4 ± 8.2	164.2 ± 9.6	0.397
Ejection Fraction	47.1 ± 13.2	47.4 ± 12.1	0.901
Troponin T	0.88 ± 2.1	0.87 ± 2.2	0.958
CK-MB ^3^	24.3 ± 62.7	16.9 ± 45.2	0.336
Fasting BST ^4^	122.6 ± 33.7	120.6 ± 33.2	0.674
BST after glucose loading	197.8 ± 57.4	203.2 ± 50.2	0.469
BST at FDG injection	191.7 ± 52.6	203.2 ± 50.2	0.363
Insulin (IU)	1.7 ± 2.1	1.7 ± 2.0	0.803
HbA1c ^5^	6.6 ± 1.5	6.8 ± 1.5	0.349
Injection-to-image time	53.1 ± 8.5	52.4 ± 7.5	0.528
Creatinine	1.4 ± 1.3	1.4 ± 1.7	0.873
Total cholesterol	155.3 ± 50.9	152.7 ± 47.6	0.699
Triglyceride	129.5 ± 80.2	146.0 ± 93.7	0.169
HDL ^6^	43.1 ± 12.7	38.9 ± 8.4	0.006 *
LDL ^7^	88.9 ± 43.9	88.2 ± 48.3	0.909

* Statistically significant; ^1^ BMI (body mass index); ^2^ MGUR (myocardial glucose uptake ratio); ^3^ CK-MB (creatinine kinase-MB); ^4^ BST (blood sugar test); ^5^ HbA1c (glycated hemoglobin); ^6^ HDL (high-density lipoprotein); ^7^ LDL (low-density lipoprotein).

## Data Availability

The data presented in this study are available on request from the corresponding author.

## Supplementary Materials

The following supporting information can be downloaded at: https://www.mdpi.com/article/10.3390/diagnostics14161705/s1, Table S1: A protocol for blood glucose maintenance (after glucose administration) to optimize uptake in cardiac FDG PET; Table S2: Myocardial glucose uptake ratio (MGUR) according to the body mass index (BMI); Figure S1: No significant MGUR difference was observed after trichotomizing patients according to body mass index (BMI) of 25 and 30.
