# Supplementary material for: Evaluation of Clinical Variables Affecting Myocardial Glucose Uptake in Cardiac FDG PET
Source: Diagnostics (Basel). 2024 Aug 6;14(16):1705. doi: 10.3390/diagnostics14161705 (PMC11353438; doi:10.3390/diagnostics14161705)

Supplementary Table S1.

A protocol for blood glucose maintenance (after glucose administration) to optimize uptake in cardiac FDG PET

Blood glucose at 45-60 min after administration	Restorative measure
160-200 mg/dL	2~4 U regular insulin IV
200~250 mg/dL	4 U regular insulin IV
> 250 mg/dL	Individually customized, based on the glucose chart review.

IV = intravenous; U = unit

Supplementary Table S2.

Myocardial glucose uptake ratio (MGUR) according to the body mass index (BMI)

BMI groups	Patients(n)	Average	Standard deviation	Median	Maximum	minimum
BMI<25	119	4.43	2.06	4.21	10.50	0.50
25≤BMI<30	77	3.79	1.71	4.00	7.81	0.58
30≤BMI	18	4.10	2.69	3.65	11.62	0.97
Total	214	4.17	2.02	4.04	11.62	0.50

Supplementary Figure S1.

No significant MGUR difference was observed after trichotomizing patients according to body mass index (BMI) of 25 and 30.

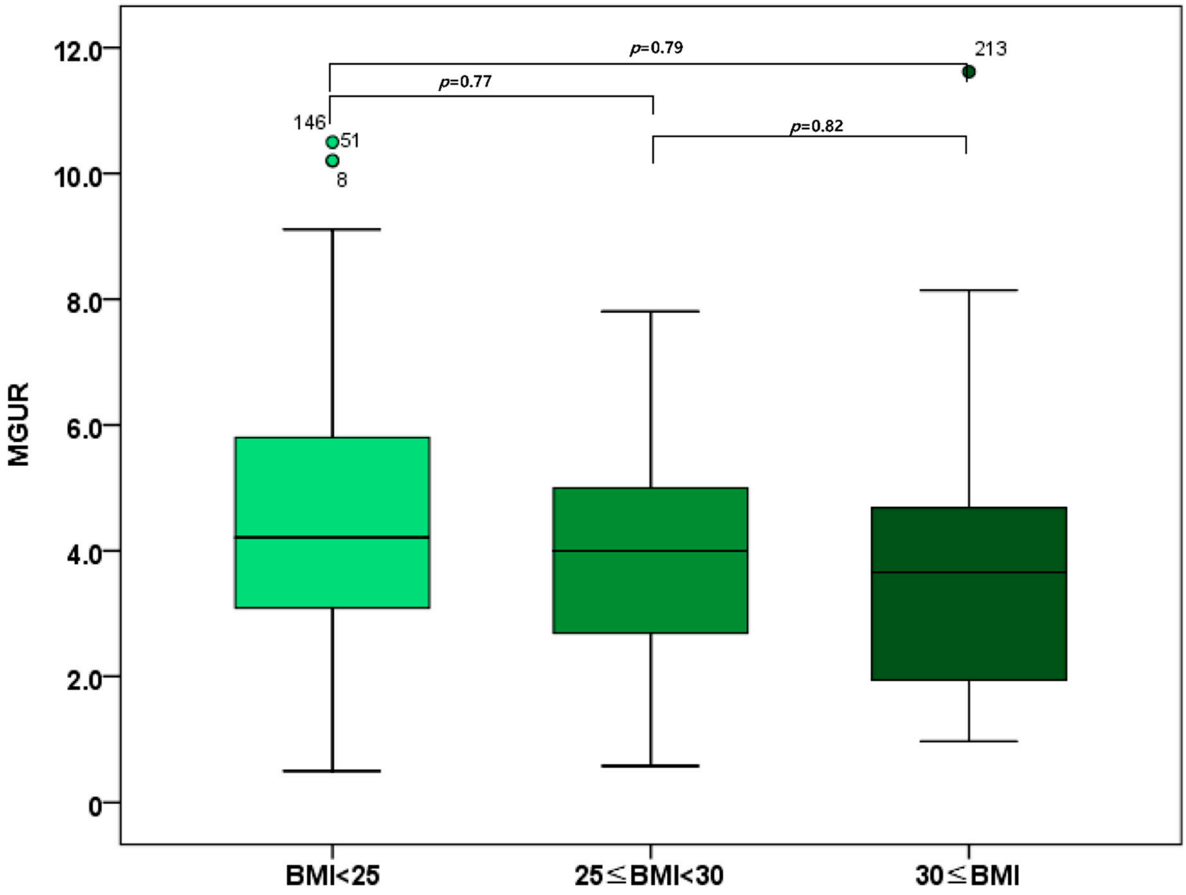

Supplement: Supplementary file 1 [file diagnostics-14-01705-s001.zip › diagnostics-3087916-supplementary.pdf]
